# Annotations

**Published:** 1909-04-03

**Authors:** 


### ANNOTATIONS.

### Deaths from Poisoning.

Some interesting returns have lately been published which embody the fatalities from the ingestion of poisons during 1907, carefully classified for purposes of comparison with previous years. Accidental deaths from scheduled poisons (those which may be retailed only by qualified chemists) numbered 142, and of these opium and its preparations caused as many as all other poisons put together. Among non-scheduled poisons ammonia and hydrochloric acid seem to do the most mischief, though their pre-eminence is not nearly so great as that of opium. With suicides, however, carbolic and oxalic acids of the scheduled poisons, and hydrochloric acid of the unscheduled, deprive opium of the chief place; but it is consolatory to learn that the addition of the first named to the schedule has been followed by a very great decline in the number of suicides effected by its aid. Hydrocyanic acid and potassium cyanide stand next to these in the favour of self-poisoners, well ahead of all other agents. Sheep-dip, vermin killer, and similar industrial poisons which the new Poisons and Pharmacy Act allows to be sold by dealers in horticultural and agricultural requisites caused remarkably few fatalities in 1907, either by accident or intent; it will be noted carefully whether any considerable increase of mortality from these poisons will attend the relaxation of these restrictions. Broadly speaking, the returns are satisfactory. Substantial declines are shown in respect of the total number of deaths from scheduled poisons, however administered; though the corresponding figures for unscheduled poisons remain about stationary. Poisonous vapours and anæsthetics show an increase, and the analysis of the cases due to the latter shows that out of 186 reported deaths, 95 were due to chloroform, seven to chloroform in mixtures with ether, ten to ether alone, two to ethyl chloride, and three to nitrous oxide gas: in 69 cases the drug used was not specified.

### Alimentary versus Aerial Infection in Tuberculosis.

In student days every medical man must have come across fellow workers who, so far from possessing conservative instincts, were usually full to the brim of some recent discovery or other, in the light of which they were ready to interpret and explain far more than ever the responsible authors had done—which, so strong is the parental pride and confidence of the human mind in its own ideas, is saying a good deal. To draw a distinction of Plato's, these comrades of our youth were imbued with philodoxy rather than with philosophy. They had a love, generous and whole hearted and well meaning, but still mischievous, of what was merely opinion. With great submission, it must be said that these reminiscences have been called forth by some passages in a speech by Sir James Crichton Browne, at a meeting convened to consider the formation of an association for prevention of consumption in Shropshire. Now, Sir James Crichton Browne has many admirable qualities as a medical publicist—scientific eminence, power of effective speaking, organising and directing ability. These attributes he has devoted to the public weal times and again. It is certain, therefore, that responsible laymen, who, to their credit, are taking expert medical advice to heart more and more every year, will be thoroughly impressed when told that practically there are just two carriers of tubercle bacilli, to wit meat and milk. It is true that a passing reference was made to inhalation, but there is no doubt that what the lay mind would lay hold of exclusively was the old warning of "death in the pot." Now this is no time or place to pronounce upon the ætiology of pulmonary tuberculosis. But it may fairly be remarked that the seemingly crucial experiments advanced by disciples of Calmette and Von Behring can be matched with the like from those thinking as Flügge and Koch do, and that there is evidence from countries like New Guinea and Japan that tubercle prevails quite independently of food infection. More than this, there are the old classic observations connecting decline in tuberculosis with improved ventilation. And it is to be noted that at the recent American Congress, Professor Woodhead did not make any sweeping deductions from the work of the British Royal Commission. It is facts like these which practically-minded laymen with large agricultural interests are ascertaining and citing, and in face of them, unduly positive statements can only check the growing faith in modern scientific medicine.

### The Action against St. Bartholomew's Hospital.

The recent action in the King's Bench Division brought by a member of the medical profession against the Governors of St. Bartholomew's Hospital for damages for personal injuries suffered by him whilst undergoing an examination under anæsthetics on the operating-table of the hospital has aroused much interest in the profession and among the managers of hospitals. The issue involved was one of much importance, and had the verdict gone the other way the consequences might have been very serious indeed to those responsible for the administration of medical charities. The plaintiff, who had entered the hospital for gratuitous treatment and examination, said that by reason of the negligence of the defendants or their servants his left arm came into contact with the hot-water tins used for heating the operating-table at the time of his examination, and that as a consequence he was paralysed in that arm, while the right arm was also injured. Defendants denied liability and said that there was no negligence on their part. They said that it was an inevitable accident, and that the injuries suffered were incident to an examination of the character made, and that the relation of master and servant did not exist between them and those engaged in the examination, and that they were not, therefore, responsible in law. After hearing most of the evidence on behalf of the plaintiff, Mr. Justice Grantham held that there was no case to go to the jury. It would be a fatal policy to the good of the country, and the injury done would be untold if he allowed it to do so, and he accordingly gave judgment for the defendants, with costs.

